# The role of non-invasive imaging modalities in cardiac allograft vasculopathy: an updated focus on current evidences

**DOI:** 10.1007/s10741-021-10155-0

**Published:** 2021-08-12

**Authors:** C Sciaccaluga, N Ghionzoli, GE Mandoli, N Sisti, F D’Ascenzi, M Focardi, S Bernazzali, G Vergaro, M Emdin, S Valente, M Cameli

**Affiliations:** 1 Department of Medical Biotechnologies, Section of Cardiology, University of Siena, Siena, Italy; 2 Department of Cardiac Surgery, University Hospital of Siena, Siena, Italy; 3 Institute of Life Sciences, Scuola Superiore Sant’Anna, Pisa, Italy; 4 Division of Cardiology and Cardiovascular Medicine, Fondazione Toscana Gabriele Monasterio, Pisa, Italy

**Keywords:** Heart transplant, CAV, Echocardiography, SPECT, CMR, CCTA

## Abstract

Cardiac allograft vasculopathy (CAV) is an obliterative and diffuse form of vasculopathy affecting almost 50% of patients after 10 years from heart transplant and represents the most common cause of long-term cardiovascular mortality among heart transplant recipients. The gold standard diagnostic technique is still invasive coronary angiography, which however holds potential for complications, especially contrast-related kidney injury and procedure-related vascular lesions. Non-invasive and contrast-sparing imaging techniques have been advocated and investigated over the past decades, in order to identify those that could replace coronary angiography or at least reach comparable accuracy in CAV detection. In addition, they could help the clinician in defining optimal timing for invasive testing. This review attempts to examine the currently available non-invasive imaging techniques that may be used in the follow-up of heart transplant patients, spanning from echocardiography to nuclear imaging, cardiac magnetic resonance and cardiac computed tomography angiography, weighting their advantages and disadvantages.

## Introduction

Cardiac allograft vasculopathy (CAV) is an obliterative and diffuse form of vasculopathy that can be considered as a late complication of heart transplant (HTx). It affects almost 50% of patients after 10 years from transplant and represents the most common cause of long-term cardiovascular mortality [1]. CAV is a distinct disease from coronary atherosclerosis, being characterized by endothelial injury [2], vascular cell proliferation, fibrosis and remodeling, and is triggered by both immune and non-immune factors. Pathophysiology of CAV is complex and not fully understood yet, involving recipient immunological response, cytomegalovirus infection, frequent episodes of acute rejection (especially antibody-mediated) and traditional cardiovascular risk factors.

Given the denervation of the cardiac allograft, CAV manifestations are often subtle, especially in early stages of disease. Invasive coronary angiography (ICA) still represents the gold standard for the diagnosis, especially when combined to intravascular ultrasound (IVUS) and optical coherence tomography (OCT) [3]. As an evidence, CAV is currently classified according to the International Society of Heart and Lung Transplantation (ISHLT) criteria, which are based on either angiographic findings and evidence of graft dysfunction, i.e., reduced left ventricular ejection fraction (LVEF) and/or restrictive filling pattern (Table 1). CAV is then graded as absent (CAV_0_), mild (CAV_1_), moderate (CAV_2_) and severe (CAV_3_), accordingly [4].

**Table 1:**
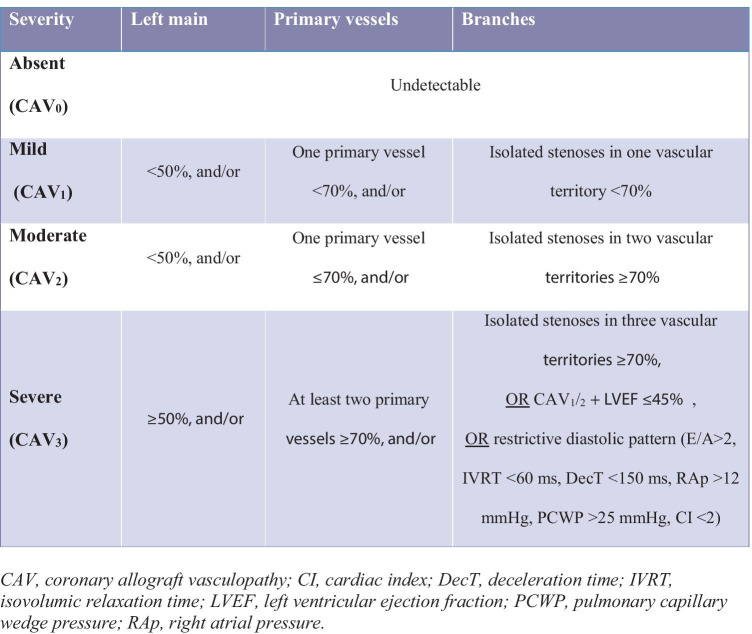
Readapted International Society of Heart and Lung Transplantation (ISHLT) classification of cardiac allograft vasculopathy

**Fig. 1:**
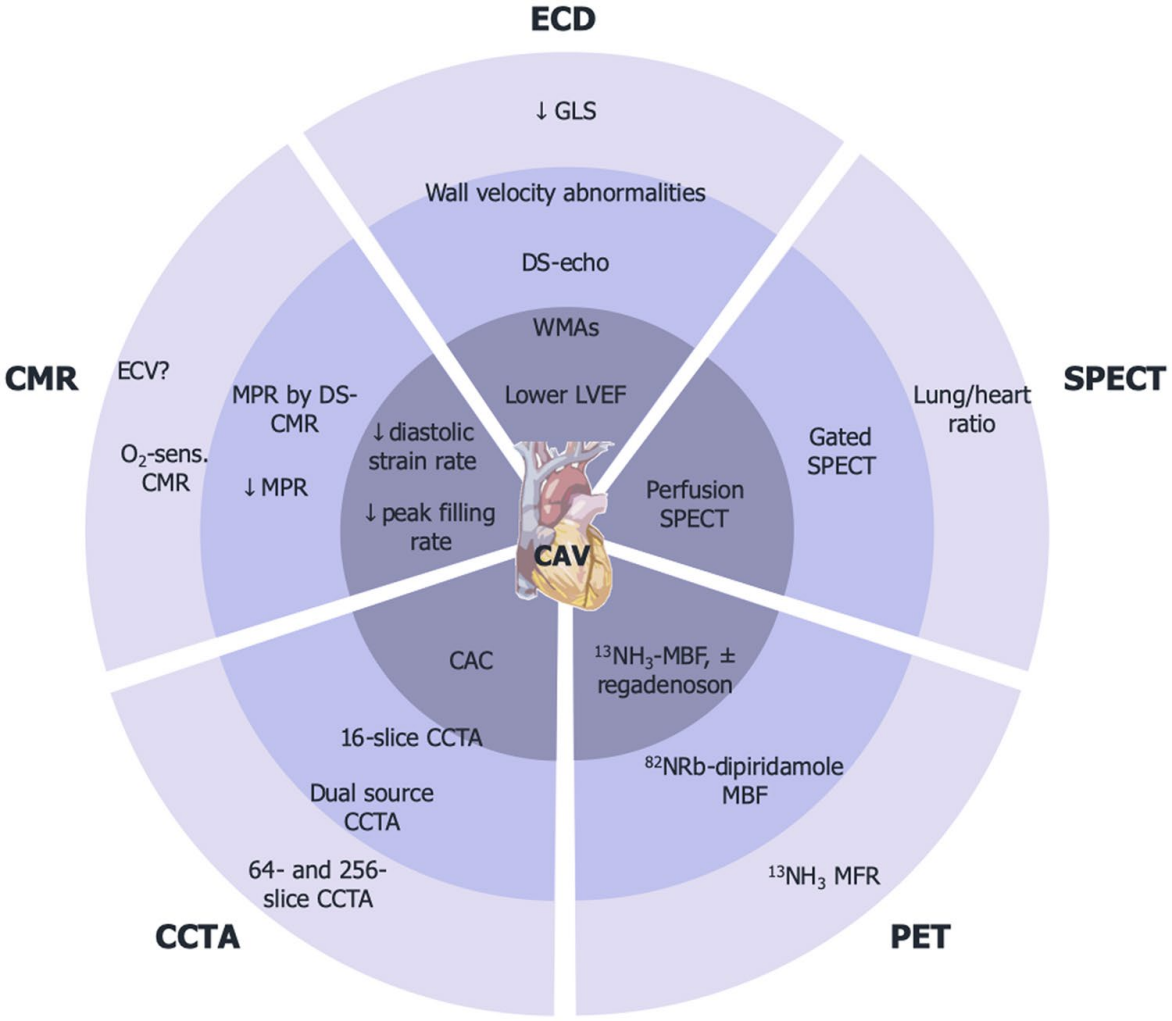
Central illustration. This figure shows the different parameters that could be assessed with each non-invasive imaging modality. The sensitivity of each parameter tends to decrease from the outer layers to the inner ones. CAC: coronary artery calcium; CAV: cardiac allograft vasculopathy; CCTA: cardiac computed tomography angiography; CMR: cardiac magnetic resonance; DS: dobutamine-stress ECD: echocardiography; GLS: global longitudinal strain: LVEF: left ventricular ejection fraction; MBF: myocardial blood flow; PET: positron emission tomography; SPECT: single-photon emission computed tomography; WMAs: wall motion abnormalities

ICA is the gold-standard method used to routinely screen HTx patients, even in the absence of left ventricular dysfunction or symptoms. However, despite its demonstrated safety, ICA holds potential for severe complications, including contrast-related kidney injury and procedure-related vascular lesions [5], with also a low sensitivity per se in detecting early CAV and a limited role in its treatment. Moreover, HTx recipients are a fragile and multi-comorbid population that often presents with renal dysfunction, mainly due to pre-HTx cardiorenal syndromes and immunosuppressive regimens [6]. Given this background, non-invasive and contrast-sparing imaging techniques have been reconsidered and investigated over the past decades, in order to identify those that could represent an alternative to ICA or at least reach comparable accuracy in CAV detection. In addition, they should provide useful information for establishing the optimal timing of ICA, further limiting its use to patients with high suspicion of CAV. This review attempts to examine the available non-invasive imaging techniques that may be used in the follow-up of HTx patients, weighting their advantages and disadvantages (Fig. 1).

## Echocardiography

### Rest echocardiography

Resting echocardiography provides limited diagnostic accuracy for CAV detection, particularly in mild forms [7–11]. The latest standardized protocol for the assessment of HTx patients includes the quantification of both diastolic and systolic function, mainly through mitral inflow Doppler velocities, LVEF and wall motion abnormalities (WMAs) [4].

#### Left ventricular ejection fraction

LVEF is often at the upper limit of normal due to either graft denervation and increased levels of circulating catecholamines, and is generally preserved even in advanced forms of CAV, making it unsuitable as an early marker of disease. David et al. showed that patients with severe forms of CAV were characterized by lower values of LVEF and a higher prevalence of grade 2 and 3 diastolic dysfunction as compared with less severe forms of disease (52% vs 62%, *p* < 0.05 and 75% vs 11%, *p* < 0.05 respectively), although LVEF was found to be preserved in the majority of them [12]. In the first years after HTx, a reduction in LVEF should be addressed more commonly to acute rejection and less commonly to CAV, while its occurrence years later might point towards CAV progression [13], stressing the role of LVEF as one of the strongest predictors of outcome in HTx [14].

#### Diastolic dysfunction

**Fig. 2:**
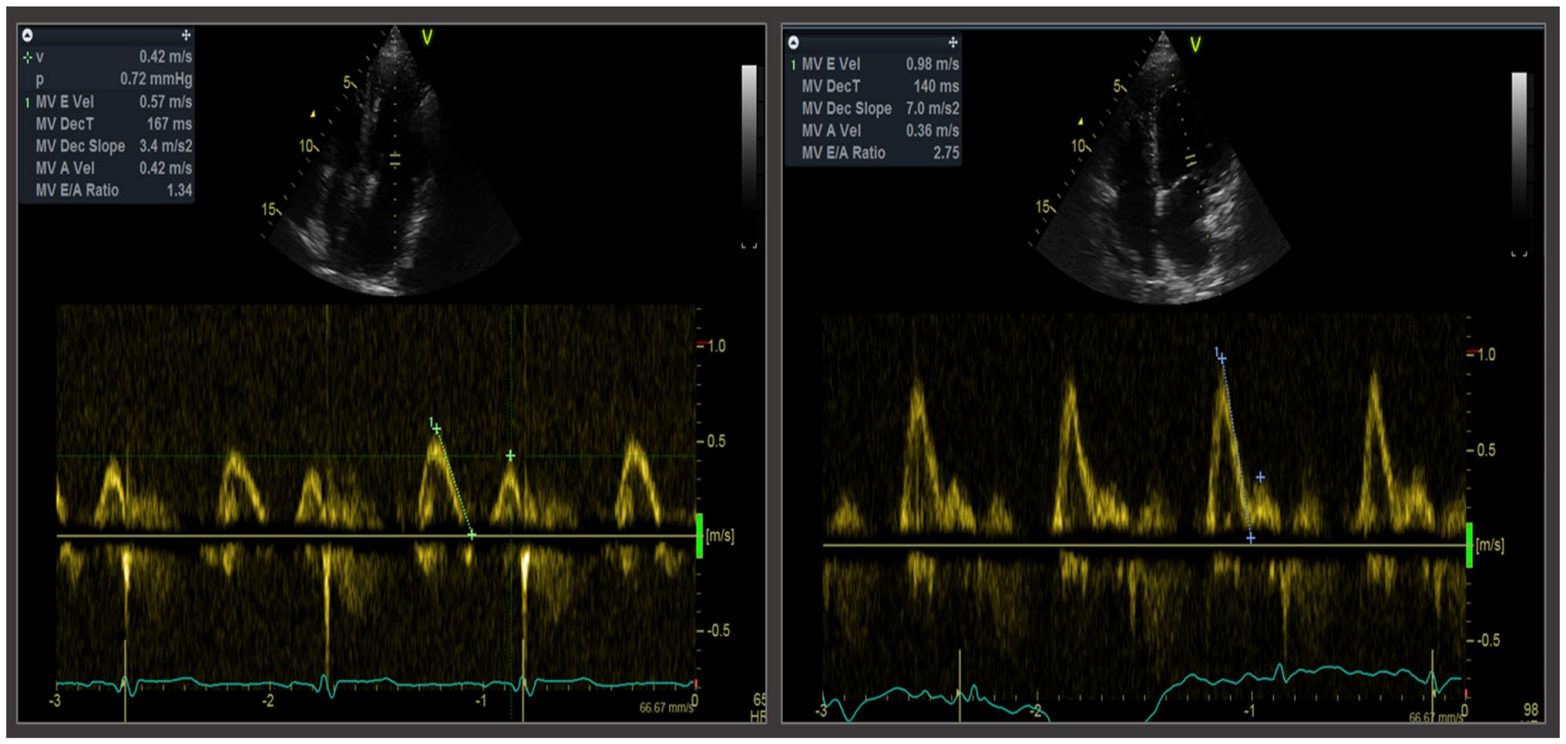
Diastolic dysfunction in presence of cardiac allograft vasculopathy. The left picture shows a normal diastolic function, as indicated by pulsed wave trans-mitral Doppler velocities and deceleration time of E wave, in a heart transplant patient without cardiac allograft vasculopathy. On the other hand, the right picture shows a restrictive diastolic pattern in presence of grade 3 cardiac allograft vasculopathy

The course of diastolic function of the transplanted heart usually presents as bimodal: in the very early phase, relevant left ventricular relaxation disturbances can be observed, even in the absence of acute rejection or CAV [7, 15, 16]; then, they attenuate after the first month. Later on, a worsening in diastolic function during follow-up should point at the possibility of acute rejection or development and/or progression of CAV (Fig. 2), deeming an invasive diagnostic approach necessary [17]. Nonetheless, this bimodal pattern was not considered in the current ISHLT classification of CAV, as a restrictive filling pattern is regarded as a grade 3 graft dysfunction, independently of the time of observation [4].

#### Wall motion abnormalities

The onset of new regional WMAs should raise suspicion towards the presence or the progression of CAV [18], thus warranting for further tests. However, these findings are not specific, as WMAs might develop even in the absence of CAV or acute rejection, especially several years after HTx [3].

Due to the development of new echocardiographic techniques, it is now possible to assess myocardial deformation, especially left ventricular (LV) global longitudinal strain (GLS) through both tissue Doppler imaging (TDI) and speckle tracking echocardiography, the latter being more angle-independent. Compared to visually assessed WMAs, wall motion velocity analysis assessed by TDI has been proven to detect earlier ventricular dysfunction [7, 19–21]. CAV patients seem to have augmented durations and reduced amplitudes of both systolic and diastolic TDI-myocardial velocities [3]. For instance, a value of radial systolic TDI-derived velocity ≤ 10 m/s showed a sensitivity of about 90% for angiographic and/or IVUS detectable CAV, but the sensitivity decreased down to 51% when investigating main epicardial vessels stenoses, even when with a 9 cm/s cut-off [18]. However, also tissue Doppler velocities at rest are more frequently indicative of advanced stages of CAV [22], and a recent study found no differences in myocardial deformation as assessed by strain analysis among normal and abnormal segments [23].

**Fig. 3:**
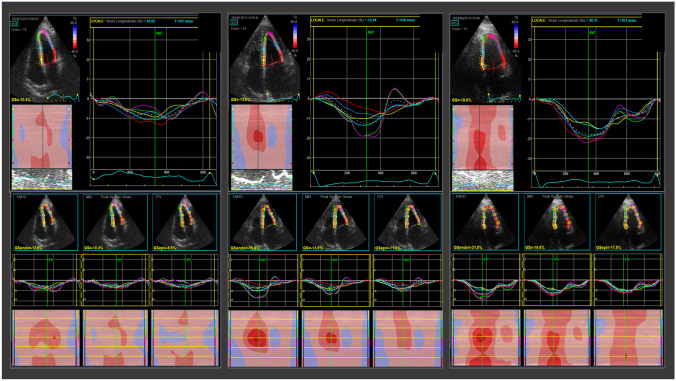
Left ventricular global longitudinal strain and cardiac allograft vasculopathy. This picture shows left ventricular longitudinal strain and three-layer specific longitudinal strain in three different heart transplant patients, with grade 3 cardiac allograft vasculopathy (CAV), with grade 1 CAV and without CAV, from left to right respectively. Left ventricular longitudinal strain assessed in apical 4-chamber view appears significantly reduced in presence of diffuse CAV, whereas it is almost comparable to normal subjects in the absence of this complication

LV-GLS is less dependent on heart rate and loading conditions as compared to LVEF and other diastolic indexes [24, 25]. The endomyocardial fibers are predominantly oriented longitudinally and represent those more susceptible to the ischemic insult, either macrovascular and microvascular. In this regard, several studies have shown a correlation between a reduced absolute value of LV-GLS and the presence of CAV and coronary microvascular dysfunction (Fig. 3) [26–28]. Clemmensen et al. found a statistically significant correlation, and a significant reduction of LV-GLS among patients with no and mild CAV (CAV_0_ vs CAV_1_), as well as a preserved LVEF even in presence of moderate-to-severe CAV [26]. Furthermore, another study attested the association of LV circumferential strain reduction to the presence of proximal coronary stenosis (positive and negative predictive value ≥ 90%, considering proximal stenosis as ≥ 50%) [29]. In addition to that, a recent study based on a small cohort of heart transplanted patients, excluding grade 3 CAV, suggested that layer-specific LV-GLS and the gradient between endocardial and epicardial longitudinal strain values could be relevant non-invasive predictors of CAV [30]. At last STE is angle-dependent and may help in the identification of LV dyssynchrony, as it can spot regional differences in LV dysfunction [20, 26, 31–33]. Indeed, patients with CAV_3_ showed a reduced absolute value of LV-GLS and a higher LV longitudinal strain time to peak, highlighting a more severe degree of LV dyssynchrony [34].

### Stress echocardiography

In the general population, exercise should be the preferred stressor when stress echocardiography is performed. Nonetheless, physical exercise might not represent an adequate cardiovascular stressor for the denervated allograft, mostly because of impaired chronotropic response [10, 35–37]. Thus, in this population of patients, stress echocardiography with pharmacological agents seems to be more favorable when this technique is indicated. However, a recent study highlighted that exercise could still represent a more efficient stressor compared to dobutamine in transplanted patients that prefer and can perform exercise stress echocardiography [38].

As concerns pharmacological agents, dobutamine stress echocardiography (DSE) represents the preferred choice [39, 40]. Unfortunately, the target heart rate may not be easily reached in some patients, and the additional use of atropine is of limited value as opposed to general population [41, 42]. In these cases, a pre-test screening with donor-recipient age difference may be considered, as it directly affects the likelihood of reaching the target heart rate [43]. Even though some studies proved good sensitivity and specificity of dipyridamole stress echocardiography in detecting CAV [39, 40], DSE still represent the first choice.

The latest ISHLT guidelines recommend dobutamine or treadmill stress echocardiography in patients who cannot undergo invasive testing (class of recommendation IIaB) [44]. In particular, these exams are routinely used in patients with or at high risk of progression of chronic kidney disease. Still, the role of DSE in the diagnosis of CAV is controversial, especially with regards to recognition of early CAV. In fact, DSE detects angiographically evident CAV [45] with a sensitivity of 70–80% that is even lower when IVUS is performed during ICA (72–79%) [46]. Furthermore, two recent studies reported a low sensitivity especially with regards to mild CAV [48, 47]. Likewise, a recent meta-analysis confirmed an insufficient sensitivity (60.2%) of DSE in the detection of CAV, despite higher specificity (85.7%) [49]. Clerkin et al. also demonstrated that DSE was not able to detect mild nor moderate CAV in the first 5 years after HTx [47].

The accuracy of stress echocardiography might improve if combined with other techniques, such as myocardial deformation analysis and the use of contrast agents. For instance, strain analysis can increase DSE sensitivity from 63 to 88% in the detection of CAV [50,51], representing a promising tool in the detection of CAV.

## Cardiac computed tomography angiography (CCTA)

Cardiac computed tomography angiography (CCTA), as most of the below-mentioned techniques, has proved useful in the diagnosis of non-allograft coronary artery disease, with the highest benefit in patients with low-to-intermediate risk of disease [52].

Regarding CAV, many concerns with the feasibility of CCTA have been raised. In particular, denervated hearts usually show elevated heart rates at rest [53]. This point, together with the common presence of impairment in renal function from multiple etiologies, may limit its use. Indeed, HTx patients are often under nephrotoxic, immunosuppressive drugs, and contrast agents may precipitate acute kidney injury. Then, if CAV is suspected, ICA becomes mandatory and further iodinated contrast is required. In spite of these issues, new modalities in CCTA (i.e., dual source, multi-segment reconstruction and motion correction algorithms) may increase its diagnostic accuracy, making this technique more appealing and useful in spite of the above-mentioned pitfalls [54].

In 2005, first evidences stated that CCTA may be used as a screening tool in HTx recipients for de novo CAV or as a follow-up strategy [55]. Furthermore, it detects an intimal maximal thickness (IMT) > 0.5 mm as well as IVUS, thus being more sensitive than ICA [56].

**Fig. 4:**
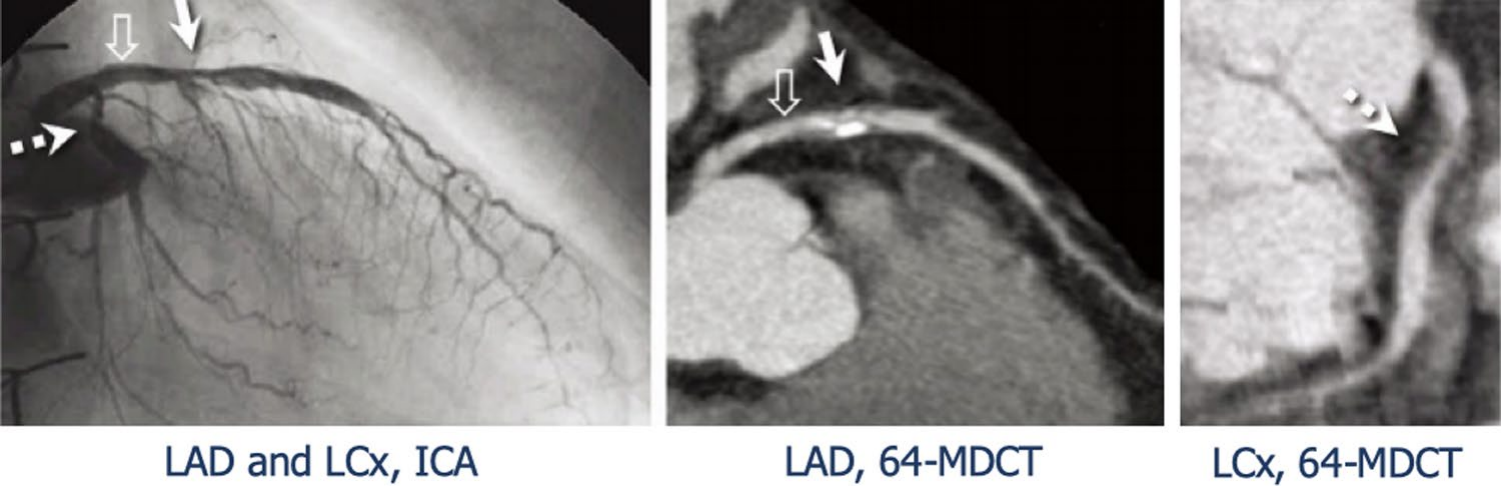
Invasive coronary angiography vs coronary computed tomography angiography. Presence of coronary allograft stenosis in proximal-middle left anterior descending artery (LAD, continuous arrows) and proximal circumflex artery (LCx, dashed arrow), as detected by either invasive coronary angiography (ICA) and 64-slice multidetector computed tomography (MDCT). ICA, invasive coronary angiography; LAD, left anterior descending artery; LCx, left circumflex artery; MDCT, multidetector computed tomography. Adapted from Nunoda S et al., 2010 (10.1253/circj.cj-09–0800) [84]

The use of multi-slice machines significantly improved the diagnostic accuracy of the technique. In a pilot study on 10 patients using 16-slice CCTA, the detection of inflammatory plaques (defined as > 30% necrosis and presence of calcium) well correlated with IVUS-derived virtual histology [57]. 64-slice was reasonably superior to 16-slice CCTA, in particular with regards to sensitivity and negative predictive value (NPV): it correctly evaluated 95% of ≥ 2 mm segments, with 100% sensitivity and NPV in finding significant, invasively treatable stenoses (> 50%) [58,59] (Fig. 4). Conversely, this technique was inaccurate in the evaluation of subtle CAV and yielded more radiation exposure than ICA (19 mSv vs. 5.7 mSv).

Whether CCTA proved overall applicable to CAV, the quantification of coronary artery calcium (CAC) showed heterogeneous results. First evidences stated that the only absence of CAC was not reliable enough to exclude CAV [60], confirming a previous study that demonstrated that CAC score was not valid to make diagnosis [61]. Instead, a high score may suggest the presence of pre-existing or de novo allograft atherosclerotic lesions. However, recent findings showed a 97% NPV in excluding moderate-to-severe CAV and 88% NPV in excluding significant stenosis at ICA [62]; patients with CAC also had more events than those without it. These data are in support of a 2012 systematic review that found 99% NPV and high sensitivity for CAC in the diagnostic workup of CAV [63].

Several parameters from CCTA—as well as their combination—may be useful in the prediction and the identification of early CAV: volume/length ratio of the plaque, wall burden and the proportion of fibrotic/fibro-fatty/calcified/low-attenuation tissue [63]. The spread of advanced technique, such as the evaluation of fractional flow reserve using computed tomography, although not tested yet in heart transplant patients, may help in defining the functional significance of a coronary stenosis, without recurring to ICA.

## Nuclear imaging

### Single-photon emission computed tomography

While the role of single-photon emission computed tomography (SPECT) is well established in the diagnosis of non-allograft coronary artery disease, its role in the diagnostic workup of CAV is way less clear. In patients with inadequate acoustic window and contraindication to contrast agents, pharmacological SPECT could represent an alternative imaging technique for CAV detection. Globally, the use of pharmacological stressors is preferred over physical exercise, and gated technique can improve sensitivity [64]. Many tracers have been tested in the context of CAV, with a solid role in prognostic stratification and a more questionable role in diagnosis.

SPECT has a high NPV, especially when combined with a normal wall motion pattern, but low specificity and sensitivity for milder cases of CAV, wherein LVEF is still preserved. This may be explained by the diffuse, balanced distribution of ischemia in CAV, as it is not territory-related [65]. In these cases, a global reduction in color-contrast may be missed, thus leading to false negatives. ^99m^Tc-tetrofosmin-gated adenosine stress SPECT was not found to be sensitive in detecting CAV, even when stricter criteria of ≥ 70% stenosis were applied [66]. Some clues may help in distinguishing between true and false negatives, in particular a lung/heart ratio > 0.37 during stress independently predict CAV even in cases of preserved LVEF, often involving main coronary branches [67]. This evidence implies that SPECT might not be ideal for early CAV detection, but it might be useful to exclude severe disease or to delay ICA, particularly in patients with previously normal epicardial coronary arteries.

As for what concerns the prognostic value of SPECT, data are more encouraging, as many studies showed that a negative stress SPECT is associated with better outcome. For instance, the presence of wall motion abnormalities together with a positive stress SPECT can predict cardiac events [67]. Manrique et al. demonstrated that a > 3 segments defect during stress could predict late revascularization at > 2 months [68]. Patients with a negative stress-SPECT with ^99m^Tc have a high NPV for major events at 12 months [69], and this trend is maintained at a 5-year evaluation [70]. Accordingly, a negative gated SPECT test holds a low risk of major cardiovascular events [68]. Large though reversible defects demonstrated to predict death [66]. In contrast, a heterogeneous uptake of tracer using SPECT showed to predict allograft dysfunction, but it was not associated with future major cardiac events and reduced survival [71].

### Positron emission tomography

The study of perfusion using positron emission tomography (PET) shows more accuracy as compared with SPECT in the diagnostic workup of non-allograft coronary artery disease [72]. This seems to apply also to the setting of CAV: the study of myocardial blood flow (MBF) can reveal the diffuse, non-segment specific nature of CAV, with earlier identification of the disease [73]. In fact, MFR assesses both macro- and microvascular function, rather than relying solely on an anatomic characterization of CAV. In the last years, growing evidence has been collected to support the use of cardiac PET in non-invasive surveillance for CAV, with recent guidelines incorporating recommendations for this technique [74].

**Fig. 5:**
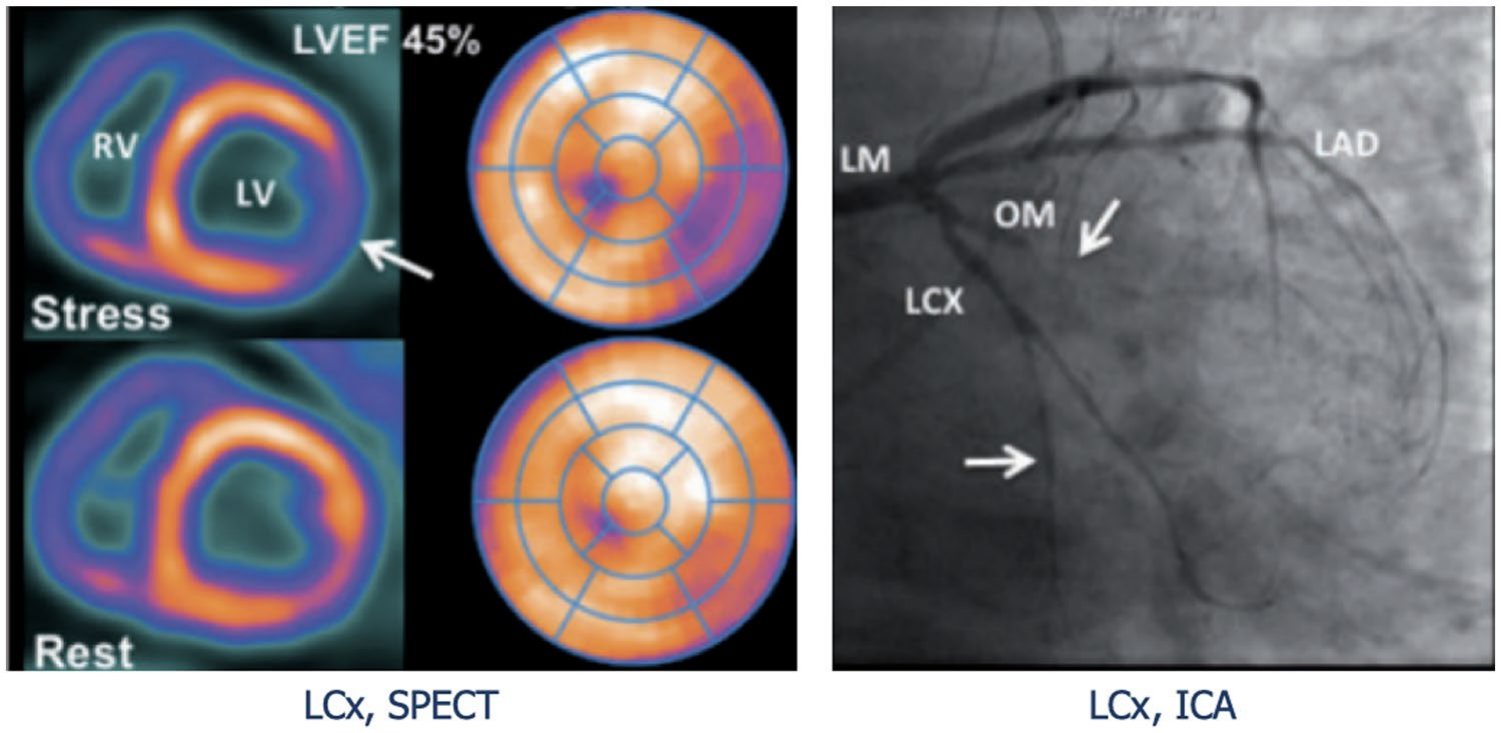
Myocardial blood flow quantification positron emission tomography. Inferolateral reduction in myocardial blood flow (MBF), suggestive of significant CAV in left circumflex artery (LCx), and milder degrees of myocardial ischemia during peak hyperemia, suggestive of diffuse CAV. ICA, invasive coronary angiography; LAD, left anterior descending artery; LCx, left circumflex artery; LM, left main artery; LV, left ventricle; LVEF, left ventricular ejection fraction; MBF, myocardial blood flow; OM, obtuse marginal; RV, right ventricle. Adapted from Bravo PE et al., 2018 (10.1093/eurheartj/ehx683) [87,76]

The first evidence comes from an outdated study by Allen-Auerbach et al., whom demonstrated that endothelial-independent MBF abnormalities were related to morphological indexes of CAV progression with IVUS [75]. ^13^NH_3_-PET with quantification of MBF provided improved detection of CAV, and valid stratification of severity, representing a strong predictor of major cardiac events [76] (Fig. 5). In a comparison study among PET, IVUS and ICA, ^82^Rb-dipiridamole PET test demonstrated that the reduction in MBF and the concurrent increase in coronary resistance were highly suggestive of CAV, with > 96% specificity [77]. Moreover, myocardial flow reserve quantification using ^13^NH_3_ was inversely related to the volume of coronary plaques as estimated by IVUS, also when ICA was negative [78]. Despite this evidence, there are not available universally accepted PET flow thresholds for CAV detection, therefore more studies are warranted in order to standardize this promising technique in the early identification of CAV in HTx patients.

PET imaging may also allow vascular wall evaluation, as a case report in 2016 demonstrated that ^18^fluorodeoxyglucose (FDG)-PET detected vascular inflammation before the onset of alterations on ICA, still using IVUS as gold standard [79]. This is line with pathophysiologic aspects of CAV, as inflammation either due to immunologic and non-immunologic factors is a leading driver of vascular impairment [80].

PET still holds prognostic information, according to several studies. Myocardial flow reserve ≤ 1.75 using ^82^Rb-dipiridamole PET was found to be related to increased rate of major cardiac events, also in the setting of preserved LVEF and normal perfusion [81]. The quantification of coronary flow reserve using the same tracer was also able to predict long-term outcome [82].

## Cardiac magnetic resonance

**Fig. 6:**
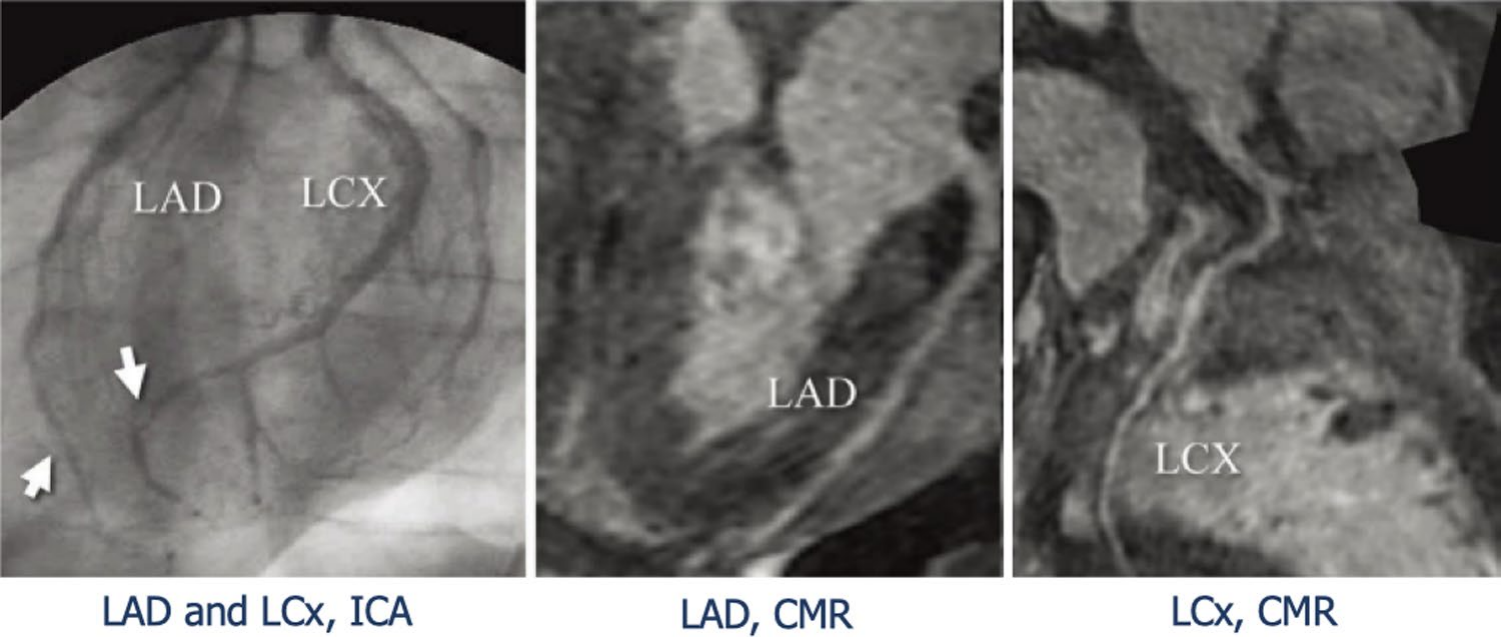
Invasive coronary angiography vs cardiac magnetic resonance. Evidence of diffuse wall thickening in the distal left anterior descending artery (LAD) and stenosis in the left circumflex artery (LCx), as revealed by invasive coronary angiography (ICA). Cardiac magnetic resonance (CMR) did not detect any of these defects. CMR, cardiac magnetic resonance; ICA, invasive coronary angiography; LAD, left anterior descending artery; LCx, left circumflex artery. Adapted from Nunoda S et al., 2010 (10.1253/circj.cj-09–0800) [84]

Cardiac magnetic resonance (CMR) represents a useful resource in the assessment of both structural and functional changes, providing also information regarding the composition of myocardial tissue. In fact, in patients with insufficient acoustic window, CMR is an alternative to echocardiography to assess cardiac chamber volumes and function as well as to exclude acute cellular rejection. In fact, its role in HTx patients has been more intensively studied with regards to acute cellular rejection, since first studies in CAV showed low sensitivity [83,85,84] (Fig. 6). First encouraging data derive from the analysis of the peak filling rate, which is an estimate of diastolic function, whose values were lower in advanced CAV rather than in earlier stages. Indeed, early diastolic strain rate impairment was successively associated to microvascular dysfunction [86], while LVEF, stroke volume and cardiac output may be found normal either in early and late stages [87]. These results might hint that diastolic dysfunction in this population might be more sensitive than indexes of systolic function for the precocious detection of CAV.

Microvascular disease can be assessed by stress perfusion CMR, through the estimate of myocardial perfusion reserve (MPR), even though first results were not encouraging in detecting CAV [84], later ones showed promising findings due to the fact that diffuse CAV can affect the microvasculature independent of lesions in the epicardial vessels. In fact, further and more detailed studies about MPR reported a lower index in those with an IMT ≥ 0.5 mm as found with IVUS, with a moderate inverse relationship [88]. MPR ≤ 1.68 has a 100% sensitivity and 100% NPV in detecting CAV, but still has low specificity (63%). This index, as well as GLS, is altered years after HTx, and should be attributable to CAV rather than to fibrosis or graft rejection [89]. Also, MPR assessed by multiparametric CMR outperformed ICA for the detection of moderate CAV in a study by Miller et al. [90].

Microvascular reactivity has also been studied by oxygenation-sensitive CMR with a protocol of 1-min hyperventilation, followed by 30 s of apnea [91]. Significant differences were found between CAV and non-CAV HTx recipients, but intriguingly this difference was maintained between mild/absent and moderate-to-severe CAV. Unfortunately, its usefulness was questioned in presence of interstitial fibrosis, but fibrosis in turn may indicate a more advanced stage of disease. This analysis might provide key information without using a stressor nor a contrast agent.

As mentioned above, contrast-enhanced CMR with gadolinium is able to identify portions of myocardium characterized by inflammation, scarring and diffuse fibrosis, which could provide valid help in serial assessment of adverse cardiac remodelling as well as strong prognostic value. Late gadolinium enhancement imaging has been widely proven to be a prognosticator in HTx patients [92], even though diffuse myocardial changes might be missed. In this context, recent techniques such as T1- and T2-mapping have been investigated in this population. Latest studies demonstrated that T2-weighted sequences can predict outcome at multivariate analysis, whilst extracellular volume and pre-contrast T1-weighted sequences tend to remain stable [93]. Perhaps, damage onset is an early finding in CAV, but tend to remain stable over time. Extracellular volume was shown to be related to vascular stenosis at ICA and to fibrosis at endomyocardial biopsy analysis; T1-relaxation time, instead, correlates only to the grade of stenosis at ICA, and IMT only to the grade of fibrosis at biopsy. With these premises, further studies should focus on whether extracellular volume is a precocious sign of CAV-induced fibrosis.

## Conclusions

The identification of CAV, especially with regard to early disease, represents a puzzling challenge for cardiologists. ICA is still the gold standard in the diagnosis of this long-term, survival-limiting disease, but does not show high sensitivity nor is free from complications. Non-invasive and more sensitive techniques are an appealing field but are still quite nebulous. To date, no technique—ICA included—is able to interpret the diffuse and subtle nature of CAV, so that such new techniques are expected to point out early alterations in vessel walls and significative early metabolic alterations for CAV. Echocardiography is a low cost, widely available technique but with limited sensitivity; the use of speckle tracking techniques may improve its diagnostic power. CCTA showed a good accuracy, but results concerning the detection of subtle CAV are controversial; the combination of multiple parameters as well as the development of sophisticated acquisitions may help in the diagnosis of earlier forms. Nuclear imaging, especially PET, will likely help in the management of HTx patients with suspected CAV, with a shift from anatomic to metabolic assessment. Nonetheless, radiation issues still limit its large-scale feasibility. MPR as assessed by CMR is promising, but anatomical vessel definition is scarce; further studies are deemed to state whether the evaluation of the extracellular volume may be an early sign of CAV. Studies regarding the combination of imaging techniques are strongly required, as their complementary nature may increase the overall sensitivity for CAV, thus limiting the need for invasive, periodic follow-ups.

## Abbreviations

CAC: Coronary artery calcium
CAV: oronary allograft vasculopathy
CCTA: Cardiac computed tomography angiography
CMR: Cardiac magnetic resonance
DSE: Dobutamine stress echocardiography
GLS: Global longitudinal strain
HTx: Heart transplant
ICA: Invasive coronary angiography
ISHLT: Nternational Society of Heart and Lung Transplantation
IMT: Intimal maximal thickness
IVUS: Intravascular ultrasounds
LVEF: Left ventricular ejection fraction
MBF: Myocardial blood flow
MPR: Myocardial perfusion reserve
NPV: Negative predictive value
PET: Positron emission tomography
SPECT: Single-photon emission computed tomography
TDI: Tissue Doppler imaging
